# Plasma proteomic analysis of a biomarker‐defined subpopulation in the SHINE Ph2 trial to identify molecular correlates to the favorable decrease in the neuroinflammatory marker GFAP with zervimesine in Alzheimer's disease participants

**DOI:** 10.1002/alz70856_107075

**Published:** 2026-01-08

**Authors:** Britney N Lizama, Valentina Di Caro, Eunah Cho, Jill Caldwell, Kiran Pandey, Duc M Duong, Nicholas T Seyfried, Michael Grundman, Anthony O Caggiano, Charlotte E. Teunissen, Mary E. Hamby

**Affiliations:** ^1^ Cognition Therapeutics, Inc., Pittsburgh, PA, USA; ^2^ Emory University School of Medicine, Atlanta, GA, USA; ^3^ Global R&D Partners, LLC, La Jolla, CA, USA; ^4^ University of California, San Diego, CA, USA; ^5^ Cognition Therapeutics, Inc., Purchase, NY, USA; ^6^ Neurochemistry Laboratory, Department of Laboratory Medicine, Amsterdam Neuroscience, Vrije Universiteit Amsterdam, Amsterdam UMC, Amsterdam, Netherlands

## Abstract

**Background:**

Participants with Alzheimer's disease (AD) treated with sigma‐2 receptor (S2R) modulator zervimesine (CT1812) exhibited 39% slowing of cognitive decline (ADAS‐Cog11) compared to placebo in the mITT cohort of the SHINE trial (NCT03507790, COG0201). The pre‐specified below‐median pTau217 subgroup showed 95% slowing with zervimesine, and significant decrease in plasma‐GFAP, a neuroinflammation biomarker, compared to placebo (‐28.35 ± 11.687 SEM, *p* = 0.0186). Given favorable outcomes with zervimesine treatment in the below‐median pTau217 subgroup, a plasma proteomic biomarker sub‐study assessing correlation of plasma proteins with plasma‐GFAP was performed to elucidate protective mechanisms of zervimesine.

**Method:**

SHINE was a Phase 2 randomized, double‐blind, placebo‐controlled study. Participants (*N* = 152) received a daily oral dose of zervimesine (100 or 300 mg) or placebo for 6‐months (end‐of‐study, EOS). A proteomics sub‐study of 60 participants was performed using tandem‐mass tag mass spectrometry (TMT‐MS) of plasma collected at baseline and EOS, with downstream analyses from treatment‐compliant participants (mITT *N* = 57). For subgroup analysis, plasma from participants with median baseline plasma *p*‐Tau217 concentration (ALZpath, SIMOA) <1pg/mL was analyzed (*N* = 24). Change from baseline (CFB) was calculated, and Pearson correlation analysis was performed with plasma‐GFAP (assessed via SIMOA) and protein levels, assessed via proteomics, from either 1) placebo and drug‐treated (all‐participants analysis) or 2) drug‐only treated participants (drug‐only analysis). Pathway analysis was performed on correlated proteins (p≤0.05).

**Result:**

TMT‐MS of plasma detected 1674 proteins, from which 45 were correlated to GFAP in the all‐participants analysis and 284 in drug‐only analysis (p≤0.05). Thirteen proteins were overlapping between the all‐participants and drug‐only analyses, including inflammation‐related proteins ALOX12 and CTSG, and lysosomal protein LAMP1. To further home in on pharmacodynamic biomarkers of zervimesine, the 13 overlapping correlates were excluded, allowing for 271 proteins specifically correlated with drug treatment to be identified (p≤0.05). These proteins were enriched in vesicle‐mediated transport and inflammation‐related pathways (FDR≤0.05).

**Conclusion:**

The correlates identified may represent an inflammatory signature tied to the decreased neuroinflammation (plasma‐GFAP) with zervimesine in AD patients observed in the pTau217 biomarker‐defined subgroup (below the median). Results support a role for zervimesine in decreasing neuroinflammation and the continued clinical development of zervimesine for AD.

